# Three‐dimensional assessment on digital cast of spontaneous upper first molar distorotation after Ni‐ti leaf springs expander and rapid maxillary expander: A two‐centre randomized controlled trial

**DOI:** 10.1111/ocr.12849

**Published:** 2024-09-08

**Authors:** Andrea Abate, Alessandro Ugolini, Alessandro Bruni, Vincenzo Quinzi, Valentina Lanteri

**Affiliations:** ^1^ Department of Sciences Integrated Surgical and Diagnostic University of Genova Genoa Italy; ^2^ Surgical, Medical and Dental Department University of Modena and Reggio Emilia Modena Italy; ^3^ Department of Life, Health & Environmental Sciences, Postgraduate School of Orthodontics University of L'Aquila L'Aquila Italy

**Keywords:** leaf expander, maxillary expansion, molar rotation, rapid maxillary expansion, transverse maxillary deficiency

## Abstract

**Objective:**

The aim of this randomized controlled trial (RCT) was to evaluate the spontaneous distorotation of upper first permanent molars and the transverse dentoalveolar changes on digital casts in growing patients following maxillary expansion treatment using either the Leaf Expander® or the rapid maxillary expander (RME), both anchored to the deciduous second molar.

**Trial Design and Setting:**

This study was a two‐arm, parallel‐assignment, RCT with a dual‐centre design conducted at two teaching hospitals in Italy.

**Participants:**

Inclusion criteria included maxillary transverse deficiency, prepubertal development stage (cervical vertebra maturation stage [CVMS] 1–2) and early mixed dentition with fully erupted upper first permanent molars. Exclusion criteria were systemic diseases or syndromes, CVMS 3–6, agenesis of upper second premolars, unavailability of the second deciduous molar for anchorage and Class III malocclusion.

**Randomization:**

Patients were randomly assigned to the Leaf Expander® or RME group using a computer‐generated randomization list created by a central randomization centre. Randomization was conducted immediately before the start of treatment.

**Intervention:**

The intervention involved treatment with either the Leaf Expander® or the RME. Both devices were anchored to the second deciduous molars. Following randomization, patients were further categorized based on the presence of no crossbite, unilateral crossbite or bilateral crossbite.

**Main Outcome Measure:**

The primary outcome measure was the distorotation of the upper first molar (U6). Secondary outcomes included measurements of interdental linear dimensions, specifically upper inter‐canine width (53–63), upper inter‐molar width (MV16–MV26) and upper inter‐deciduous second molar width (55–65).

**Blinding:**

The examiner analysing the digital casts was blinded to the treatment groups to prevent detection bias and ensure objective assessment. However, due to the nature of the intervention, blinding was not feasible for the patients and clinicians involved in administering the treatment.

**Results:**

A total of 150 patients were enrolled and randomly assigned to two groups: 75 to the Leaf Expander® group and 75 to the RME group. Recruitment started in November 2021 and was completed in November 2022. At the time of analysis, the trial was complete with no ongoing follow‐ups. ANOVA tests revealed no significant differences between the three subgroups (no‐cross, unilateral‐cross and bilateral‐cross) within both the Leaf Expander® and RME groups at T0. The Leaf Expander® demonstrated significantly greater distorotation in the unilateral crossbite subgroup compared to the RME (*p* = .014). In terms of total molar distorotation, the Leaf Expander® appliance showed a significantly greater effect (12.66°) compared with conventional RME (7.83°). Linear regression analysis demonstrated a significant correlation between the extent of expansion and the degree of molar rotation.

**Conclusions:**

Maxillary expansion resulted in significant spontaneous molar distorotation when the appliance was bonded to the second deciduous molars. The Leaf Expander® exhibited significantly greater molar distorotation compared with conventional RME. The degree of molar distorotation was correlated with the extent of expansion obtained on the second deciduous molar.

**Trial Registration:**

The trial was registered at ClinicalTrials.gov (ID: NCT05135962).

## 1 INTRODUCTION

The transverse deficiency of the upper jaw represents one of the most frequently encountered issues in orthodontics. This results in a malocclusion that can be clinically accompanied with a mono‐ or bilateral crossbite.^1^

Literature have suggested that this condition occurs in 8%–20% of children.^2^, ^3^

The aetiology of the transverse skeletal deficiency of the maxilla can be attributed to primary maxillary hypoplasia, where reduced dimensions are linked to hereditary‐constitutional factors, or secondary, when caused by detrimental habits such as non‐nutritive thumb sucking, excessive use of dummies and baby bottles, nasal respiratory insufficiency, oral breathing or low tongue posture.^4^

The likelihood of spontaneous self‐correction of the defect following cessation of etiological factors, such as certain bad habits, is low (0% to 9%).^5^, ^6^

Palatal expansion is facilitated during childhood while the mid‐palatal suture remains fibrous and poorly interdigitated, up until the complete ossification of circummaxillary sutures.^7^, ^8^, ^9^

Literature describes different appliances capable of expanding the upper jaw and various expansion protocols. The primary distinction is made between removable and fixed appliances.^10^

Based on the activation protocol, slow, semi‐rapid and rapid expansion are distinguished according to the speed of expansion.^11^

The Leaf Expander is a nickel‐titanium device designed to apply uniform, gradual and continuous force. Its primary benefits include straightforward activation and independence from patient cooperation, thus eliminating compliance issues.^12^, ^13^ Additionally, compared to conventional RME, the Leaf Expander typically results in lower levels of pain during the initial days following its application.^14^

Regarding the spontaneous distorotation of first permanent molars following different modalities of maxillary expansion, the literature is currently notably lacking. The only study available to date is conducted by Cerruto et al.,^15^ demonstrating that, following an interceptive phase of RME, there is a spontaneous distorotation of upper first permanent molars. Additionally, spontaneous distorotation of first permanent molars seems to be particularly higher when deciduous second molars are used as anchorage.

This clinical result can translate into a significant increase in upper arch length, a potential improvement in Class II malocclusion.^16^, ^17^

To date, no clinical study has been conducted to evaluate the spontaneous distorotation of first permanent molars following rapid and slow maxillary expansion. Understanding the differences in the outcomes of these two treatment methods is crucial for optimizing orthodontic interventions in growing patients. The Leaf Expander® and rapid maxillary expander (RME) are commonly used devices, yet their comparative effects on molar distorotation remain unclear. Although there is some literature on their impact on dentoalveolar changes, the specific aspect of spontaneous molar distorotation has not been thoroughly investigated. Therefore, the objective of this research was to conduct a randomized clinical study using secondary data analysis from a two‐centre randomized controlled trial, specifically evaluating the spontaneous distorotation of first permanent molars and the associated dentoalveolar changes after palatal expansion with the Leaf Expander and RME. This study aims to fill the gap in the literature and provide evidence‐based guidance for clinicians. The study's primary endpoint was to test the null hypothesis H0, which posits that there are no significant differences in first upper molar distorotation measurements following treatment with the Leaf Expander compared to the RME.

## 2 MATERIALS AND METHODS

### 2.1 Trial design and study registration

The present study is a randomized controlled trial (RCT) with a two‐arm parallel assignment and a dual‐centre design. The protocol followed guidance from the CONsolidated Standards of Reporting Trials (CONSORT) Guidelines.^18^ The study was conducted at the Department of *Hidden* (centre 1#) and at the Orthodontics Department **Hidden**(centre 2#). The study was registered before its initiation on ClinicalTrials.gov with the following identifier: **Hidden**.

The study received approval from the Ethics Committee of the of the **Hidden**. All procedures performed in this RCT involving human participants were in accordance with the ethical standards of the institutional and/or research committee and with the 1964 Helsinki declaration and its later amendments or comparable ethical standards.

### 2.2 Participants and study settings

In this study, patients referred to the coordinating centre 1 and to the centre 2 were recruited from November 2021 and November 2022.

The inclusion criteria were as follow:

- Caucasian origin,
- prepubertal developmental stage (cervical vertebral maturation stage [CVMS] 1 or 2),^19^
- Mixed dentition with well‐preserved deciduous second molars to use as anchorage. The second deciduous molar was considered available as anchoring tooth when the relative upper second premolar cusps was positioned apically to half pulp chamber (HPC) line of the ipsilateral upper first permanent molars on pre‐treatment panoramic radiographs,^20^
- Fully erupted permanent upper first molars,
- Posterior transverse discrepancy of at least 3 mm. It was calculated on the digital dental models, by calculating the difference between maxillary intermolar width (distance between central fossae of permanent maxillary first molars right and left) and mandibular intermolar width (distance between distobuccal cusp tips of permanent mandibular first molars right and left).^21^ This may encompass patients with crossbite (unilateral or bilateral) or without crossbite.

The exclusion criteria adopted were:

- Presence of multiple and/or advanced caries,
- Presence of supernumerary teeth,
- Previous orthopaedic/orthodontic treatment,
- Pubertal or postpubertal stage of development (CVMS 3–6),
- Agenesis of upper second premolars,
- Patients with documented pathologies (congenital deformities or acquired pathologies) such us cleft lip and/or palate and craniofacial syndromes or patients with Obstructive Sleep Apnea Syndromes (OSAS).
- Patients older than 12 years,
- Patients presenting skeletal class III malocclusion.

All parents of the patients provided informed consent prior to the commencement of the trial.

### 2.3 Intervention

**FIGURE 1:**
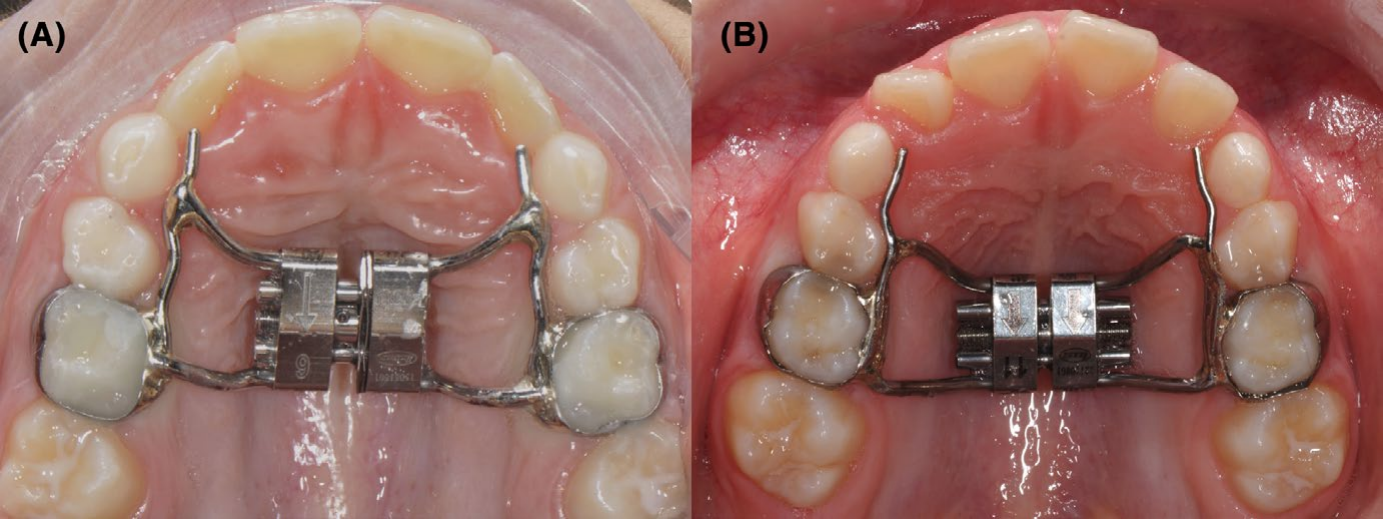
(A) Leaf expander cemented on the second deciduous molars; (B) Rapid maxillary expander (RME) cemented on the second deciduous molars.

Subjects assigned to the RME group underwent maxillary expansion using a RME (Figure 1A). The RME was fixed to the upper second deciduous molars using bands and includes a midline 12‐mm self‐locking screw (Leone, Sesto Fiorentino, Italy).

The expansion protocol was one quarter‐turn twice a day (0.45 mm activation per day) until overcorrection.

At the time of appliance delivery, written and verbal oral hygiene instructions were given, including cleaning methods. Also, written informed consent was obtained from each patient or the parents.

Subjects in the Leaf expander group underwent maxillary expansion using the Leaf Expander® (Leone, Sesto Fiorentino, Italy), a device fixed to the upper second deciduous molars using bands (Figure 1B). This expander features a double nickel‐titanium leaf spring design, activated by turning a central chrome‐cobalt steel screw, compressing two or more nickel‐titanium leaf springs.

The activation protocol involved selecting the maximum expansion quantity (in mm) based on the patient's transverse discrepancy (6 or 9‐mm screw), utilizing a force of 900 g. Each activation of the central screw produced 0.1 mm of expansion, requiring 10 activations for 1 mm.

The procedure for maxillary expansion utilizing the Leaf Expander entailed an initial pre‐activation of the device to attain a 3/4.5 mm expansion, typically accomplished within a span of 2–3 months. Subsequently, clinicians proceeded to reactivate the device during in‐office appointments by manipulating the leaf springs, implementing 10/15 quarter turns to the screw monthly until the desired expansion was reached.

In each protocol, maxillary expansion was conducted until achieving dental overcorrection, characterized by the lingual cusps of the upper first permanent molars occluding onto the buccal cusps of the lower first permanent molars, as delineated by Caprioglio et al.^22^

Both Leaf Exanders and RME were left passively for retention for a minimum of 6 months.

Following a 12‐month duration, both the Leaf expanders and the RME devices were removed, and during this interval, none of the patients underwent supplementary orthodontic interventions.

Each expander exhibited a 0.9 mm stainless steel wire framework lingually to the maxillary deciduous canines, lacking posterior extension to the maxillary permanent first molars.

Two clinicians treated the patients in centre #1 and in the centre #2. The clinical experience was similar for all clinicians (5–10 years of experience in orthodontics).

### 2.4 Outcomes

The stereolithographic (.STL) files obtained from the same extraoral scanner were imported into the reverse modelling software package Mimics Materialize® 26.0 (Materialize, Leuven, Belgium) to perform all measurements by two operators (A.A. and A.B.). Each study cast scan was manually pre‐processed to remove unwanted data artefacts from the analysis. The digital models were analysed using protocols previously described in literature for defining landmarks and measures.^12^, ^15^, ^23^

**FIGURE 2:**
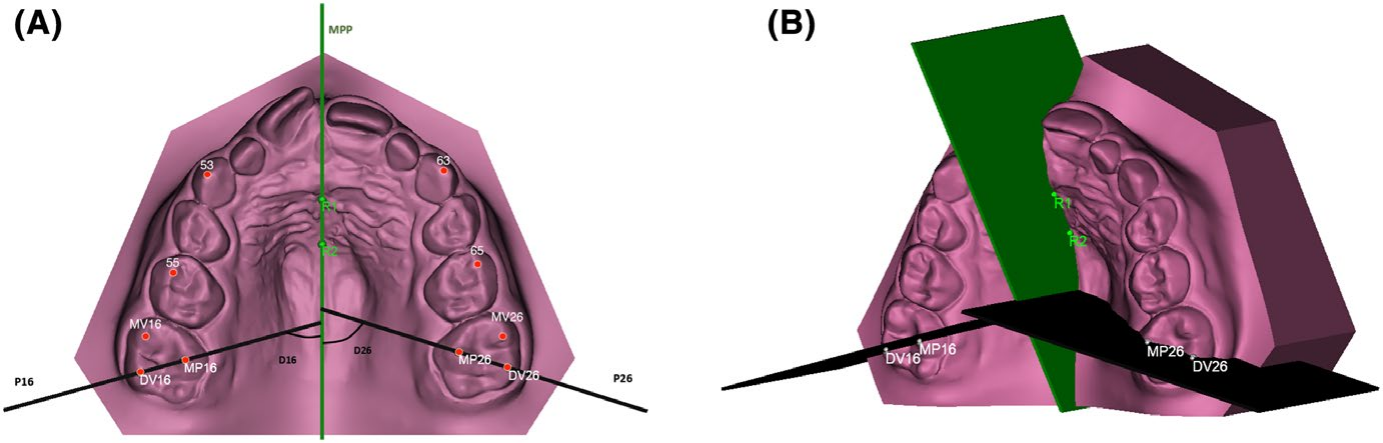
(A) The median palatal plane (MPP) was delineated by connecting two landmarks identified along the median palatal raphe, illustrated in green. The initial landmark pinpointed the location on the median palatal raphe adjacent to the second ruga (point R1). Subsequently, the second landmark (point R2) was positioned on the median palatal raphe, 1 cm distal to the first point. P16 plane passing through DV16 and MP16, and P26 plane passing through DV26 and MP26.; (B) MPP, P16 and P26 plans for the evaluation of the distorotation of the first permanent molars in three‐dimensional view.

In the digital model of the upper dental arch, specific landmarks have been defined as follows (Figure 2A):

- R1 and R2: Two points, one anterior and one posterior, situated at the level of the median palatal raphe. The initial landmark pinpointed the location on the median palatal raphe adjacent to the second ruga (point R1). Subsequently, the second landmark (point R2) was positioned on the median palatal raphe, 1 cm distal to the first point.
- 53 and 63: Tips of the deciduous right and left canine teeth.
- 55 and 65: Mesio‐vestibular cusps of the deciduous right and left second molars.

At the level of the permanent first molars on the right (16) and left (26), the following points have been defined:

- DV16 and DV26: Disto‐vestibular cusps.
- MV16 and MV26 Mesio‐vestibular cusp.
- MP16 and MP26: Mesio‐palatal cusps.

The primary outcome was to evaluate on the upper first molar (U6) distorotation defined as the angle between the line passing through the distobuccal‐mesiopalatal cusps and the MPP.

To assess the extent of distorotation of the permanent first molars, the following planes have been identified (Figure 2B):

- MPP (mid palatal plane): Median palatal plane passing through R1 and R2, perpendicular to the base plane of the digital model.^23^
- P16: Plane passing through DV16 and MP16.
- P26: Plane passing through DV26 and MP26.

Angular measurements, denoted as D16 and D26, were then recorded, representing the angles formed by the intersection of the MPP with the planes P16 and P26, respectively.

The secondary outcome was to evaluate the inter‐dental linear measurements, defined as follow:

- Upper inter‐canine width (53–63): measured at the cusp level as the distance between the cusp tips of right and left maxillary canines;
- Upper inter‐molar width (MV16–MV26): measured at cusp level as the distance between the mesiobuccal cusp tips of right and left maxillary first molars;
- Upper inter‐deciduous second molar width (55–65): measured at the cusp level as the distance between the cusp tips of right and left maxillary deciduous second molars;
- The two observers (A.A. and A.B.) carried out all measurements three times.

### 2.5 Sample size

The G*Power free software (version 3.1.9.4, Franz Faul, Universitat Kiel, Kiel, Germany) was initially used to obtain data for the power analysis calculation. Prior to the commencement of the current study, a sample size calculation was performed with the aim of determining the number of patients required to detect statistically significant differences between the two treatment modalities.

In this study, the sample size was determined based on the upper first molar distorotation as the primary outcome variable.

As there were no existing data in the scientific literature, the mean and standard deviation of a preliminary sample of 15 patients (RME δ = 7.56°, RME σ = 4.84; Leaf δ = 11.06°, Leaf δ = 5.27) were considered, with a a two‐tailed significance level of 5% and target power of 80% and 20% beta error level. The analysis indicated that a minimum of 68 patients, 34 patients in each group, were required considering a 10% withdrawal rate from the study. The sample analysed was part of another randomized clinical study, the sample calculation reporting that the sample is sufficient to detect the difference in the distortion of the first permanent molars (secondary data analysis).

### 2.6 Randomization and allocation concealment

Patients who fulfilled the eligibility criteria were enrolled and randomly allocated into the two groups using the Microsoft Excel (Microsoft, Redmond, WA, USA) random number generator. Block and stratified randomization techniques were employed to allocate an equivalent number of patients to each treatment group across the two centres.

The allocation sequence was safeguarded from bias using an opaque and sealed envelope, each sequentially numbered for the respective centres. These envelopes remained unopened until the operators were ready to prepare the expander for cementation.

### 2.7 Blinding

The orthodontists who performed the treatment were not aware of the randomization procedure, and therefore into which group the patients had been assigned, but due to clear differences in the design of the appliance, it was not possible to keep him blinded even during the treatment period. The measurements calculated on the digital models were performed by two operators (A.A. and A.B.) who was not aware of the treatment performed by the various patients as the operators did not know to which group the patients had been assigned.

### 2.8 Statistical analysis

The statistical analysis of the collected data was performed using SPSS for Windows software (version 23.0; SPSS, Chicago, IL). Descriptive statistics were employed to analyse the data. The Shapiro–Wilk test was utilized to assess normal distribution of the data. Since the data demonstrated a normal distribution, statistical analysis was conducted using parametric tests. A baseline comparison between the two groups, Leaf Expander and RME, was executed via independent samples *T*‐test to ascertain the homogeneity of the two groups.

The net difference between T0 and T1 were compared among the three different subgroups (No‐cross, Unilateral‐Cross, Bilateral‐Cross) through one‐way analysis of variance (ANOVA) test. Subsequent pairwise comparisons were conducted using Tukey post hoc test with Bonferroni correction.

Independent samples *T*‐tests were utilized to compare the net differences obtained through Leaf Expander and RME for each variable in the three subgroups.

Furthermore, the total amount of rotation (D16 + D26) achieved by the two devices was compared using an independent samples *T*‐test between Leaf Expander and RME, considering the sum of distorotation of 16 and 26. The test was also conducted by comparing the rotation occurred on all analysed molars, thus doubling the sample size and enhancing the statistical significance of the test.

Finally, a linear regression model was employed to evaluate the correlation between deciduous maxillary expansion magnitude (55–65) and amount of permanent molar distorotation (D16 + D26).

All measurements were performed by the same operators (A.A. and A.B.). Method error assessment was conducted by repeating measurements of 20 randomly selected cases by the first operator and a second operator after a 15‐day interval. Intra‐class correlation coefficient (ICC) test was adopted to assess intra‐operator and inter‐operator reliability. Dhalberg's formula was also calculated to assess the random error.^24^

The significance level for both ANOVA and *T*‐tests was set at 0.05.

## 3 RESULTS

**FIGURE 3:**
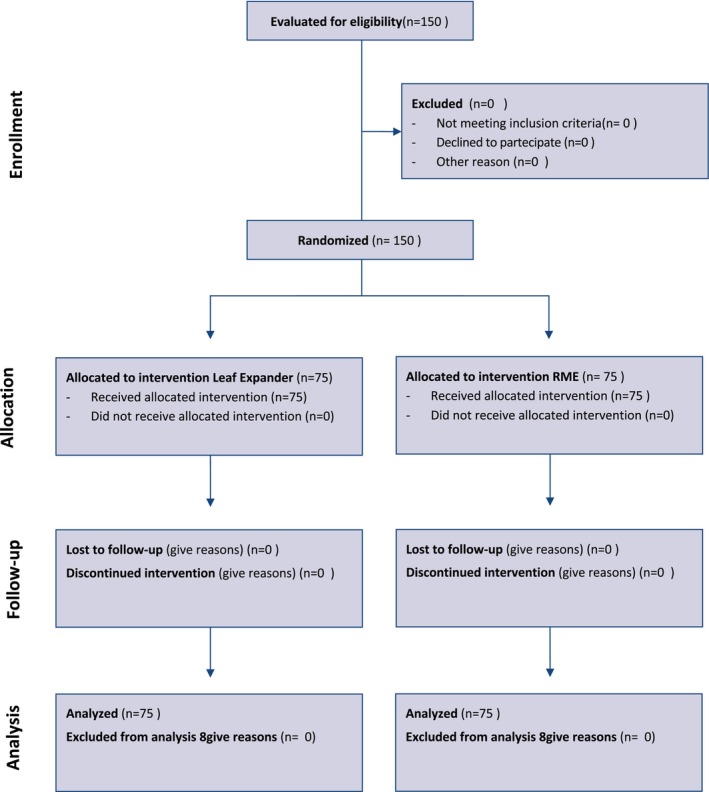
Consort flow diagram. Da: Schulz et al. [25].

A total of 150 patients were enrolled in the trial and randomly assigned to undergo maxillary expansion using two types of screws. Seventy five patients were assigned to the Leaf expander group (Leaf Group) and 75 patients were assigned to the conventional RME (Figure 3).

The two groups were then subdivided into the following subgroups: subjects without crossbite (No‐Cross), subjects with unilateral crossbite (Unilateral‐Cross) and subjects with bilateral crossbite (Bilateral‐Cross) with the aim to evaluate the influence of the posterior crossbite on the upper first molar distorotation.

The Leaf Expander group consisted of 32 males and 43 females aged between 6 and 10 years (mean age: 7.84 ± 0.5). The composition was as follows: 31 subjects with no crossbite, 27 presenting unilateral crossbite (16 left and 11 right) and 17 bilateral crossbite.

The RME group consisted of 35 males and 40 females aged between 6 and 10 years (mean age: 7.68 ± 0.92). The composition was as follows: 30 subjects with no crossbite, 30 presenting unilateral crossbite (16 left and 14 right) and 15 bilateral crossbite.

Recruitment and treatment of patients and the relative follow‐up occurred at Orthodontic Department of the two University involved, between November 2021 and November 2023.

All patients adhered to their randomly assigned treatments.

There were no dropouts during the trial, and the protocol remained unaltered (see Figure 3).

The intra‐observer and inter‐observer reproducibility ICC (average ± SD, range) demonstrated excellent results for all variable: 0.987 ± 0.018, 0.937–0.995 and 0.968 ± 0.017, 0.931–0.984 respectively. Moreover, according to Dahlberg's formula, the random error for linear measurements was about 0.13 mm and 0.23° for angular measurements.

The Shapiro–Wilk test demonstrated that the collected data exhibited a normal distribution. Table 1 highlighted that there were no statistically significant differences among the three subgroups at T0 between the Leaf Expander and RME, exhibiting a similar position at time T0 of the variables considered in this study.

**TABLE 1:** Descriptive statistics of study cohort (*n* = 150), presenting mean values along with their respective standard deviations (SD), were computed for the pre‐treatment comparisons between the Ni‐Ti leaf springs expander (Leaf Expander) group (*n* = 75) and rapid maxillary expansion (RME) group (*n* = 75). Independent sample *t*‐tests were subsequently utilized to compare the two groups.

	No‐ cross T0 versus T0			Unilateral T0 versus T0			Bilateral T0 versus T0		
	Leaf expander (*n* = 31)	RME (*n* = 30)	Significance	Leaf expander (*n* = 27)	RME (*n* = 30)	Significance	Leaf expander (*n* = 17)	RME (*n* = 15)	Significance
	Mean ± SD	Mean ± SD	*p* value	Mean ± SD	Mean ± SD	*p* value	Mean ± SD	Mean ± SD	*p* value
53–63 (mm)	30.56 ± 2.65	30.09 ± 2.45	.46	30.06 ± 1.45	29.48 ± 1.62	.07	30.20 ± 1.67	28.83 ± 1.67	.05
55–65 (mm)	41.96 ± 2.05	41.02 ± 2.62	.18	42.35 ± 2.62	40.34 ± 1.52	.05	39.06 ± 2.77	39.1 ± 1.82	.95
16–26 (mm)	48.23 ± 1.76	46.62 ± 2.54	.06	47.22 ± 2.6	46.32 ± 2.14	.16	44.62 ± 3.31	45.02 ± 1.13	.63
D16 (°)	79.84 ± 5.92	81.2 ± 4.85	.33	77.48 ± 6.15^a^	79.76 ± 5.15^a^	.13	83.69 ± 4.75	80.9 ± 4.07	.05
D26 (°)	77.48 ± 5.74	76.15 ± 5.52	.36	78.52 ± 6.51^b^	79.9 ± 5.5^b^	.39	77.54 ± 5.26	77.81 ± 4.05	.86

*Note*: Bold—significant difference between groups (*p* value < .05).

^a^ Cross bite side for unilateral patients.

^b^ No cross bite side for unilateral patients.

The mean treatment duration in the LE group was 8 ± 3 months. in the RPE group, the mean active treatment duration was 10 ± 2 days, and the total treatment duration was 9 ± 1 months. The average number of appointments was 6 ± 2 in the LE group and 8 ± 1 in the RPE group.

Regarding the ANOVA test for comparing the three subgroups treated with RME, a significantly greater increase(*p* = .002) in inter‐canine and deciduous inter‐molar distance was observed in the Bilateral‐crossbite subgroup compared to the Unilateral‐crossbite and No‐crossbite subgroups. No significant differences emerged among the subgroups concerning the distorotation of the first permanent molars (Table 2).

**TABLE 2:** Comparison of the changes between the three subgroups after RME and Leaf expander by means of One‐way analysis of variance (ANOVA) test and Tukeys post‐hoc with Bonferroni's correction.

Variables	Leaf expander T1–T0 changes			RME T1–T0 changes		
	No Cross (*n* = 31)	Unilateral (*n* = 27)	Bilateral (*n* = 17)	No Cross (*n* = 30)	Unilateral (*n* = 30)	Bilateral (*n* = 15)
	T1–T0 Mean ± SD	T1–T0 Mean ± SD	T1‐–T0 Mean ± SD	T1‐T0 Mean ± SD	T1–T0 Mean ± SD	T1–T0 Mean ± SD
53–63 (mm)	5.36 ± 2.01	5.20 ± 1.61	5.68 ± 1.60	4.02 ± 2.01	3.42 ± 1.43	4.71 ± 1.33
55–65 (mm)	5.52 ± 1,39	5.41 ± 1.99	7.16 ± 1.17	4.47 ± 1.57	4.22 ± 1.54	5.23 ± 1.37
16–26 (mm)	3.42 ± 1.16	4.60 ± 1.43	5.44 ± 1.68	4.13 ± 1.77	4.26 ± 1.86	4.64 ± 1.33
D16 (°)	−6,24 ± 3,52	−6.91 ± 3.89^a^	−6.97 ± 3.75	−4.17 ± 2.84	−3.63 ± 2.54^a^	4.50 ± 2.45^a^
D26 (°)	−5,90 ± 3,26	−6.68 ± 5.11^b^	−5.10 ± 2.94	−4.08 ± 2.10	−4.35 ± 3.01^b^	2.71 ± 1.8^b^

One‐way analysis of variance (ANOVA)					
	53–63	55–65	16–26	D16	D26
RME					
ANOVA test *p* value	**.026**	**.046**	NS	NS	NS
No versus Unilat	0.154	0.531	0.773	0.955	0.418
No versus Bilat	**0.036**	**0.043**	0.242	0.740	0.083
Unilat versus Bilat	**0.002**	**0.015**	0.386	0.789	0.287
Leaf expander					
ANOVA test *p* value	NS	**.001**	**.001**	NS	NS
No versus Unilat	0.790	0.810	**0.001**	0.457	0.573
No versus Bilat	0.328	**0.001**	**0.001**	0.885	0.376
Unilat versus Bilat	0.483	**0.001**	0.099	0.602	0.210

*Note*: Bold—significant difference between groups (p value<.05).

^a^ Cross bite side for Unilateral patients.

^b^ No cross bite side for Unilateral patients.

The ANOVA test for the Leaf Expander group instead highlighted a significant increase in deciduous intermolar distance (*p* < .01) and permanent intermolar distance (*p* < .01) in the Bilateral‐crossbite subgroup compared to the No‐Crossbite and Unilateral‐crossbite subgroups. No significant differences were noticed among the subgroups in the D16 and D26 variables (Table 2).

From the statistical analysis comparing the net differences between T0‐T1 for the RME and Leaf expander group, a statistically significant difference was observed for most of the variables. Specifically, all variables considered except for the distance 16–26 showed a statistically significant difference in favour of the Leaf Expander, as reported in Table 3.

**TABLE 3:** Comparison of the changes occurred after Ni‐Ti leaf springs expander (Leaf) and rapid maxillary expansion (RME) assessed with independent sample *t*‐test.

	No‐ cross difference T1–T0			Unilateral difference T1–T0			Bilateral T0 versus T0		
	Leaf expander	RME	Significance	Leaf expander	RME	Significance	Leaf expander	RME	Significance
	Mean ± SD	Mean(SD)	*p* value	Mean ± SD	Mean ± SD	*p* value	Mean ± SD	Mean ± SD	*p* value
53–63 (mm)	5.36 ± 2.02	4.02 ± 2.01	**.01**	5.20 ± 1.61	3.42 ± 1.43	**<.01**	5.68 ± 1.60	4.71 ± 1.33	**.05**
55–65 (mm)	5.52 ± 1.39	4.47 ± 1.57	**.008**	5.41 ± 1.99	4.22 ± 1.54	**.014**	7.16 ± 1.17	5.23 ± 1.37	**<.01**
16–26 (mm)	3.42 ± 1.16	4.13 ± 1.77	.07	4.60 ± 1.43	4.26 ± 1.86	.43	5.44 ± 1.68	4.64 ± 1.33	.12
D16 (°)	−6.24 ± 3.52	−4.17 ± 2.84	**.014**	−6.91 ± 3.89^a^	−3.63 ± 2.54^a^	**<.01**	−6.97 ± 3.75	4.50 ± 2.45	**.027**
D26 (°)	−5.91 ± 3.26	−4.08 ± 2.10	**.012**	−6.68 ± 5.11^b^	−4.35 ± 3.01^b^	**.044**	−5‐.10 ± 2.94	2.71 ± 1.8	**.007**

*Note*: Bold—Significant difference between groups (*p* value < .05).

^a^ Cross bite side for monolateral patients.

^b^ No cross bite side for monolateral patients.

More specifically, considering the Unilateral‐crossbite subgroups and the Bilateral‐crossbite subgroup, the Leaf Expander demonstrated greater efficacy in the spontaneous distorotation of the first permanent molars on the side affected by cross‐bite compared to RME. This result was also achieved through two additional comparisons: (1) by considering 16 and 26 separately (D6) for each group with the aim of doubling the sample size and increasing the reliability of the statistical test (Table S1); (2) by summing the amount of distorotation (D16 + D26) of the right and left first permanent upper molars in the RME and Leaf Expander group group (Table S1).

Additionally, despite a comparable initial situation, all molars presenting cross‐bite and no cross‐bite were grouped together, and a comparison of the extent of distorotation between the two expansion modalities was performed (Table S1).

The Leaf Expander group has proven to be more effective in producing distorotation of the first permanent molar for all variables considered (Table S1).

Concerning the linear regression models, in the RME group (Figure S1A), no significant correlation was observed (*p* = .71) with an *R* ^2^ = .65. However, in the Leaf group (Figure S1B), a statistically significant correlation between the two variables was observed (*p* = .0086) with an *R* ^2^ = .85. These results demonstrated that an increase in maxillary expansion at the level of the deciduous second molars (55 + 65) produced by the Leaf expander is correlated with an increase in the distorotation of the permanent molars (D16 + D26).

## 4 DISCUSSION

This randomized controlled trial demonstrated spontaneous distorotation of the upper first permanent molars among growing patients treated with both the Leaf Expander and RME when anchored to the deciduous second molars. In addition, the study confirmed the ability of both treatments to modify the interdental width of the upper arch.

Maxillary expansion represents the treatment of choice for addressing transverse maxillary deficiency, solving unilateral or bilateral crossbite and increasing the total length of the upper arch.^26^, ^27^, ^28^ However, its effect on the distorotation of the upper first permanent molars remain unclear.

The aim of this two‐centre randomized clinical trial (RCT) was to assess and compare the spontaneous upper first molar distorotation and the dento‐alveolar effects produced by the Leaf Expander® with those of RME, on digital dental casts. Notably, this study stands as the first RCT to take into consideration this parameter after maxillary expansion comparing the effect produced by the Leaf expander and the RME.

In the present study, it was observed that following maxillary expansion with anchorage on the deciduous second molars, there is a spontaneous distorotation of the first permanent molars. This finding is consistent with data reported in the only RCT published in literature that evaluate the effect of RME on the upper first molar distorotation.^15^ The authors highlighted that the first maxillary molars distorotated significantly more when deciduous teeth were used as anchorage instead of the first permanent molars. This phenomenon could be attributed to the triangular opening of the mid‐palatal suture resulting from the positioning of the centre of resistance of the maxilla in relation to the screw position,^29^, ^30^ which would result in spontaneous distorotation of the permanent molars. Furthermore, the upper first molars are free to move and to adapt to the new occlusal situation as they are not banded.

In this study, deciduous teeth were utilized as anchoring teeth, following the recommendation of different authors,^7^, ^26^, ^31^, ^32^ who highlighted how the utilization of deciduous teeth as anchorage can mitigate potential periodontal and endodontic damage associated with RME on permanent anchoring teeth.

In this study, no statistically significant difference between the two groups was found at baseline (T0) for the variables suggesting a reduction in biases when comparing the two cohorts.

Concerning the subgroups analysis with ANOVA test, the results showed that no significant difference was found for distorotation among the subgroups that utilized the same device. Analysing the dento‐alveolar variables a greater expansion in subjects presenting bilateral crossbite was found compared with the other two subgroups.

Comparing the two expansion protocols, no statistically significant difference was noticed for the T0–T1 net difference in upper inter‐molar width variable (MV16–MV26). Conversely, despite a comparable maxillary deficit between the two groups at T0 and a similar activation quantity of the devices, a greater dento‐alveolar expansion of the maxillary arch in terms of intercanine distance (53–63) and deciduous intermolar distance (55–65) was observed in the Leaf group compared to the RME group.

These results disagree with those reported by Paoloni et al.,^33^ in which a significant greater inter‐canine (53–63) expansion was found in the RME group. It must be specified that in the research mentioned above the authors used butterfly‐shaped stainless‐steel framework which did not include extensions up to the deciduous canines, differently to the appliance design used in the present study. The results of the present study agree with a retrospective study by Cossellu et al.^34^ performed on digital dental casts who found that deciduous inter‐canine width (53–63) was significantly greater in the Leaf Expander group. These favourable results for the leaf expander are due to the greater dentoalveolar effect exerted on the anchoring deciduous teeth. However, the device achieves a similar effect compared to the RME on the permanent teeth with only minimal differences at the skeletal level.^12^

Recently, Abate et al.^12^ corroborate the effectiveness of Leaf Expander and RME in patients during mixed dentition by means of a CBCT examination. The author reported similar skeletal and dento‐alveolar effects between the two expansion modalities with difference in the skeletal variables lower than 1 mm and irrelevant from the clinical point. This data is in agreement with those previously published by Paoloni et al.^33^ who reported no significant difference between the RME and Leaf expander® groups for any of the dento‐skeletal variables and less than 1.5 mm of difference in the skeletal Mx‐Mx variable.

In the current study, the Leaf Expander® showed a statistically significant greater upper first molar spontaneous distorotation compared to the RME group. Furthermore, it was observed that within the subgroups of Unilateral‐crossbite and Bilateral‐crossbite, the Leaf Expander® demonstrated superior efficacy in inducing spontaneous molar distorotation in subjects presenting posterior cross‐bite compared to RME.

Moreover, despite initially comparable conditions, all molars with and without cross‐bite were pooled together for comparison of distorotation extent between Leaf Expander® and conventional RME. The Leaf Expander® group consistently exhibited superior effectiveness in inducing spontaneous distorotation of the upper permanent molars both in the absence or presence of posterior cross‐bite.

The clinical significance of these findings is that the Leaf Expander® may be a more effective choice for inducing molar distorotation, especially in patients with posterior cross‐bite. The greater distorotation achieved with the Leaf Expander® could potentially lead to better treatment outcomes in certain malocclusions, providing clinicians with a valuable alternative to RME.

The reason why the Leaf Expander® induces greater distorotation of the first permanent molars is due to the protocol of device activation, which involves the use of light and continuous forces, on the contrary RME is based on heavy and intermittent forces. In fact, the linear regression model revealed a highly significant correlation in the Leaf Expander® group between the amount of distorotation on the first permanent molar and the expansion achieved on the deciduous second molars. It appears that the upper first molars, influenced by the transseptal fibres, follow the movement of the deciduous second molars, as already reported in the literature,^35^ promoting both transverse expansion and simultaneously causing rotation in a mesiodistal direction. This change may be attributed to the stretching of the transseptal fibres positioned between the deciduous second molar and the first permanent molar, which are mesial to the latter and its corresponding centre of resistance, resulting in spontaneous movement of distorotation.

Indeed, this spontaneous effect may potentially occur during conventional RME as well, but to a lesser extent. This is because the hyalinization process of the periodontal ligament, induced by the use of heavy forces, considerably reduces the movement of deciduous teeth.^36^ In fact, it is known that in this expansion protocol, distorotation is primarily due to the triangular opening of the mid‐palatal suture.^15^ Conversely, the light and continuous forces generated by the Leaf Expander® would induce slightly less skeletal expansion, as highlighted in the literature,^12^, ^33^ but a greater dentoalveolar effect, especially at the level of the anchoring deciduous teeth, as demonstrated in the present study. This would justify a greater stretching of the fibres and the consequent spontaneous movement of the permanent molars. In a study by Tenshin et al.,^37^ the mechanisms of remodelling of the transseptal fibres during tooth movement and retention period were analysed. It was demonstrated that the remodelling mechanism of the transseptal fibres depends on the degree of force applied to the tooth. Likely, the light and continuous forces of the Leaf Expander® allow the transseptal fibres to adapt more easily to the new position of the teeth after maxillary expansion compared to the heavy forces of rapid expansion. Further studies will be needed to investigate the biological and biomechanical principles underlying this type of movement.

### 4.1 Strengths and limitations of the study

In terms of strengths, the study design featured innovation by being conducted in two different centres, thereby enhancing the generalizability of the findings. Furthermore, the research demonstrated a high level of reliability for each dentoalveolar variable, with both intra‐observer and inter‐observer reliability exceeding 0.90, and a standard error that can be considered negligible. Additionally, to the best of our knowledge, only one randomized clinical trial (RCT) has evaluated these parameters following maxillary expansion, and none have incorporated the Leaf Expander® device into their protocol.

A major strength of this study is the adherence to an Intention to Treat (ITT) protocol design. This approach ensures that all participants who were initially randomized were included in the analysis, preserving the integrity of the randomization process. Notably, the zero drop‐out rate further underscores the robustness of the study's findings.

Based on the results from two different clinical centres, our findings are likely to be generalizable to a broader population. However, additional research involving a larger number of institutions and varied patient populations would further support the generalizability of these findings. Moreover, increasing the number of subjects involved would also be beneficial.

Among the limitations of this study, we can mention the absence of follow‐up observations after removing the appliance. Nonetheless, the authors have planned to collect follow‐up data at least 1 year after expander removal in order to evaluate the stability of the results.

## 5 CONCLUSION

The results of this study indicate that both treatments are effective in modifying the upper dental arch dimensions in growing patients. This randomized controlled trial assessed the spontaneous distorotation achieved after expansion performed with the Leaf Expander® compared to that achieved with Rapid Maxillary Expander. Taken together, the results of this study demonstrate the following:

- A significant increase in maxillary dental arch transverse dimensions was observed in both groups, with no statistically significant differences between them.
- Maxillary expansion achieved with both RME and Leaf Expander® induces spontaneous molar distorotation when the appliance is anchored to the deciduous teeth.
- The Leaf Expander® group showed a significantly greater amount of upper first molar distorotation compared to the RME group, particularly in cases of posterior cross‐bite.

In light of the discussions, this study reinforces the importance of anchoring expansion devices, when possible, to the deciduous second molars. Based on these findings, we can speculate that the application of light and continuous forces, as provided by the Leaf Expander®, may contribute to more effective molar distorotation, which in some cases can aid in achieving the goals of orthodontic treatment in growing patients.

## AUTHOR CONTRIBUTIONS

**Conceptualization:** A.A., V.L. and A.U.; **Methodology**: A.A., A.B. and A.U.; **Investigation:** A.A., V.Q. and A.U.; **Validation:** A.A. and A.U.; **Software**, A.A., A.U. and A.B.; **Formal Analysis**: V.L.; **Data Curation:** A.A. and A.U.; **Writing—Original Draft Preparation**: A.A., A.B. and V.Q.; **Writing—Review And Editing:**, A.A., A.B, V.L. and A.U.; **Supervision**: A.U. and V.L. All authors have read and agreed to the published version of the manuscript.

## CONFLICT OF INTEREST STATEMENT

The authors have declared no conflicts of interest. The authors certify that, no financial support or benefits have been received by any coauthor, by any member of their immediate families or by any individual or entity with whom or with which they have a significant relationship from any commercial source that is related directly or indirectly to the scientific work reported in this article.

## ETHICAL APPROVAL

The study was conducted at the Department of Biomedical Surgical and Dental Sciences, IRCCS Cà Granda Foundation, University of Milan (centre 1#) and at the Orthodontics Department of the University of Genoa (centre 2#). The study was registered before its initiation on ClinicalTrials.gov with the following identifier: NCT05135962. The study received approval from the Ethics Committee of the of the Fondazione IRCCS Ca'Granda, Ospedale Maggiore, Milan ‐ Italy (No. 51/2021 dated 18.05.21). All procedures performed in this RCT involving human participants were in accordance with the ethical standards of the institutional and/or research committee and with the 1964 Helsinki declaration and its later amendments or comparable ethical standards.

## INFORMED CONSENT

For this type of study, informed consent was obtained from all parents' patients or their guardians.

## Supporting information


Figure S1.

Table S1.

## Data Availability

The data underlying this article will be shared on reasonable request to the corresponding author.

## References

[1] Lione R , Franchi L , Cozza P . Does rapid maxillary expansion induce adverse effects in growing subjects? Angle Orthod. 2013;83(1):172‐182. 22827478 10.2319/041012-300.1 PMC8805530

[2] Thilander B , Wahlund S , Lennartsson B . The effect of early interceptive treatment in children with posterior cross‐bite. Eur J Orthod. 1984;6(1):25‐34. doi:10.1093/EJO/6.1.25 6583062

[3] Ciuffolo F , Manzoli L , D'Attilio M , et al. Prevalence and distribution by gender of occlusal characteristics in a sample of Italian secondary school students: a cross‐sectional study. Eur J Orthod. 2005;27(6):601‐606. doi:10.1093/EJO/CJI043 16009668

[4] Nieri M , Paoloni V , Lione R , et al. Comparison between two screws for maxillary expansion: a multicenter randomized controlled trial on patient's reported outcome measures. Eur J Orthod. 2021;43(3):293‐300. doi:10.1093/ejo/cjaa063 33215652

[5] Viggiano D , Fasano D , Monaco G , Strohmenger L . Breast feeding, bottle feeding, and non‐nutritive sucking; effects on occlusion in deciduous dentition. Arch Dis Child. 2004;89(12):1121‐1123. 15557045 10.1136/adc.2003.029728 PMC1719762

[6] Kennedy DB , Osepchook M . Unilateral posterior crossbite with mandibular shift: a review. J Can Dent Assoc. 2005;71(8):569. 16202196

[7] Maschio M , Gaffuri F , Ugolini A , Lanteri V , Abate A , Caprioglio A . Buccal alveolar bone changes and upper first molar displacement after maxillary expansion with RME, Ni‐Ti leaf springs expander and tooth‐bone‐borne expander. A CBCT based analysis. Eur J Paediatr Dent. 2023;24(3):211‐215. 37668460 10.23804/ejpd.2023.1896

[8] Petrén S , Bondemark L , Söderfeldt B . A systematic review concerning early orthodontic treatment of unilateral posterior crossbite. Angle Orthod. 2003;73(5):588‐596. 14580028 10.1043/0003-3219(2003)073<0588:ASRCEO>2.0.CO;2

[9] McNamara JA , Lione R , Franchi L , et al. The role of rapid maxillary expansion in the promotion of oral and general health. Prog Orthod. 2015;16:1‐7. 26446931 10.1186/s40510-015-0105-x PMC4596248

[10] Leonardi R , Sicurezza E , Cutrera A , Barbato E . Early post‐treatment changes of circumaxillary sutures in young patients treated with rapid maxillary expansion. Angle Orthod. 2011;81(1):36‐41. 20936952 10.2319/050910-250.1 PMC8926357

[11] Perillo L , De Rosa A , Iaselli F , d'Apuzzo F , Grassia V , Cappabianca S . Comparison between rapid and mixed maxillary expansion through an assessment of dento‐skeletal effects on posteroanterior cephalometry. Prog Orthod. 2014;15:1‐8. 25139110 10.1186/s40510-014-0046-9 PMC4138550

[12] Abate A , Ugolini A , Maspero C , Silvestrini‐Biavati F , Caprioglio A , Lanteri V . Comparison of the skeletal, dentoalveolar, and periodontal changes after Ni–Ti leaf spring expander and rapid maxillary expansion: a three‐dimensional CBCT based evaluation. Clin Oral Investig. 2023;27(9):5249‐5262. 10.1007/s00784-023-05144-6 PMC10492880 37466717

[13] Lanteri V , Abate A , Cavagnetto D , et al. Cephalometric changes following maxillary expansion with Ni‐Ti Leaf Springs palatal expander and rapid maxillary expander: a retrospective study. Appl Sci. 2021;11(12):5748. doi:10.3390/app11125748

[14] Ugolini A , Cossellu G , Farronato M , Silvestrini‐Biavati A , Lanteri V . A multicenter, prospective, randomized trial of pain and discomfort during maxillary expansion: leaf expander versus hyrax expander. Int J Paediatr Dent. 2020;30(4):421‐428. 31894603 10.1111/ipd.12612

[15] Cerruto C , Ugolini A , Vece L , Doldo T , Caprioglio A , Silvestrini‐Biavati A . Cephalometric and dental arch changes to Haas‐type rapid maxillary expander anchored to deciduous vs permanent molars: a multicenter, randomized controlled trial. J Orofac Orthop. 2017;78(5):385‐393. 28397083 10.1007/s00056-017-0092-2

[16] Lione R , Paoloni V , De Razza FC , Pavoni C , Cozza P . The efficacy and predictability of maxillary first molar Derotation with Invisalign: a prospective clinical study in growing subjects. Appl Sci. 2022;12(5):2670. doi:10.3390/app12052670

[17] de Oliveira VC , da Rocha VE , Junior LRM , Paranhos LR , Ramos AL . Rotation of the upper first molar in class I, II, and III patients. Eur J Dent. 2016;10(1):59‐63. doi:10.4103/1305-7456.175696 27011741 PMC4784155

[18] Moher D , Hopewell S , Schulz KF , et al. CONSORT 2010 Explanation and elaboration: updated guidelines for reporting parallel group randomised trials. BMJ. 2010;340:741. 10.1136/bmj.c869 PMC2844943 20332511

[19] Baccetti T , Franchi L , McNamara JA Jr . An improved version of the cervical vertebral maturation (CVM) method for the assessment of mandibular growth. Angle Orthod. 2002;72(4):316‐323. 12169031 10.1043/0003-3219(2002)072<0316:AIVOTC>2.0.CO;2

[20] Quinzi V , Federici Canova F , Rizzo FA , Marzo G , Rosa M , Primozic J . Factors related to maxillary expander loss due to anchoring deciduous molars exfoliation during treatment in the mixed dentition phase. Eur J Orthod. 2021;43(3):332‐337. 33215659 10.1093/ejo/cjaa061

[21] Tollaro I , Baccetti T , Franchi L , Tanasescu CD . Role of posterior transverse interarch discrepancy in class II, division 1 malocclusion during the mixed dentition phase. Am J Orthod Dentofacial Orthop. 1996;110(4):417‐422. 8876494 10.1016/s0889-5406(96)70045-8

[22] Caprioglio A , Bergamini C , Franchi L , et al. Prediction of class II improvement after rapid maxillary expansion in early mixed dentition. Prog Orthod. 2017;18:1‐8. 28367605 10.1186/s40510-017-0163-3 PMC5376539

[23] Leonardi R , Lo Giudice A , Rugeri M , Muraglie S , Cordasco G , Barbato E . Three‐dimensional evaluation on digital casts of maxillary palatal size and morphology in patients with functional posterior crossbite. Eur J Orthod. 2018;40(5):556‐562. 29474543 10.1093/ejo/cjx103

[24] Dhalberg G . Statistical methods for medical and biological students. Br Med J. 1940;2(4158):358‐359.

[25] Schulz FK , Altman DG , Moher D , CONSORT Group . CONSORT 2010 Spiegazione ed Elaborazione: linee guida aggiornate per il reporting di trial randomizzati a gruppi paralleli. Evidence. 2012;4(7):e1000024.

[26] Agostino P , Ugolini A , Signori A , Silvestrini‐Biavati A , Harrison JE , Riley P . Orthodontic treatment for posterior crossbites. Cochrane Database Syst Rev. 2014;8:CD000979. 10.1002/14651858.CD000979.pub2 25104166

[27] Bucci R , D'Antò V , Rongo R , Valletta R , Martina R , Michelotti A . Dental and skeletal effects of palatal expansion techniques: a systematic review of the current evidence from systematic reviews and meta‐analyses. J Oral Rehabil. 2016;43(7):543‐564. 27004835 10.1111/joor.12393

[28] Ugolini A , Abate A , Donelli M , et al. Spontaneous mandibular dentoalveolar changes after rapid maxillary expansion (RME), slow maxillary expansion (SME), and leaf expander—a systematic review. Children. 2024;11(4):501. doi:10.3390/children11040501 38671718 PMC11049362

[29] da Silva Filho OG , do Prado Montes LA , Torelly LF . Rapid maxillary expansion in the deciduous and mixed dentition evaluated through posteroanterior cephalometric analysis. Am J Orthod Dentofacial Orthop. 1995;107(3):268‐275. 7879759 10.1016/s0889-5406(95)70142-7

[30] Davidovitch M , Efstathiou S , Sarne O , Vardimon AD . Skeletal and dental response to rapid maxillary expansion with 2‐versus 4‐band appliances. Am J Orthod Dentofacial Orthop. 2005;127(4):483‐492. 15821693 10.1016/j.ajodo.2004.01.021

[31] Brunetto M , Andriani J d SP , Ribeiro GLU , Locks A , Correa M , Correa LR . Three‐dimensional assessment of buccal alveolar bone after rapid and slow maxillary expansion: a clinical trial study. Am J Orthod Dentofacial Orthop. 2013;143(5):633‐644. 23631965 10.1016/j.ajodo.2012.12.008

[32] Bazargani F , Lund H , Magnuson A , Ludwig B . Skeletal and dentoalveolar effects using tooth‐borne and tooth‐bone‐borne RME appliances: a randomized controlled trial with 1‐year follow‐up. Eur J Orthod. 2021;43(3):245‐253. 32761047 10.1093/ejo/cjaa040

[33] Paoloni V , Giuntini V , Lione R , et al. Comparison of the dento‐skeletal effects produced by leaf expander versus rapid maxillary expander in prepubertal patients: a two‐center randomized controlled trial. Eur J Orthod. 2022;44(2):163‐169. 34114608 10.1093/ejo/cjab035

[34] Cossellu G , Ugolini A , Beretta M , et al. Three‐dimensional evaluation of slow maxillary expansion with leaf expander vs. rapid maxillary expansion in a sample of growing patients: direct effects on maxillary arch and spontaneous mandibular response. Appl Sci. 2020;10(13):4512. doi:10.3390/app10134512

[35] Rosa M , Lucchi P , Manti G , Caprioglio A . Rapid palatal expansion in the absence of posterior cross‐bite to intercept maxillary incisor crowding in the mixed dentition: a CBCT evaluation of spontaneous changes of untouched permanent molars. Eur J Paediatr Dent. 2016;17(4):286‐294. 28045316

[36] Bruni A , Abate A , Maspero C , Castroflorio T . Comparison of mechanical behavior of clear aligner and rapid palatal expander on transverse plane: an in vitro study. Bioengineering. 2024;11(2):103. doi:10.3390/bioengineering11020103 38391589 PMC10886082

[37] Tenshin S , Tuchihashi M , Sou K , et al. Remodeling mechanisms of transseptal fibers during and after tooth movement. Angle Orthod. 1995;65(2):141‐150. 7785805 10.1043/0003-3219(1995)065<0141:RMOTFD>2.0.CO;2

