# Supplementary figures and images for: Three‐dimensional assessment on digital cast of spontaneous upper first molar distorotation after Ni‐ti leaf springs expander and rapid maxillary expander: A two‐centre randomized controlled trial
Source: Orthod Craniofac Res. 2024 Sep 8;28(1):104–15. doi: 10.1111/ocr.12849 (PMC11701968; doi:10.1111/ocr.12849)

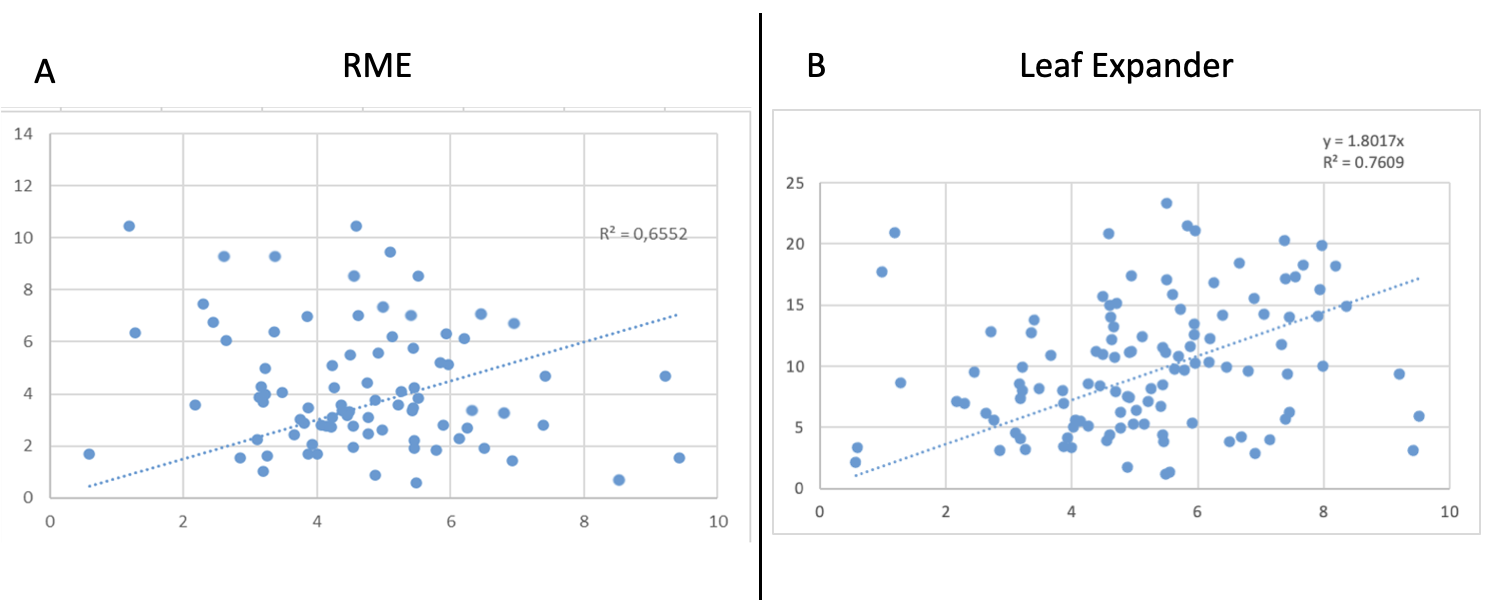

Supplement: Supplementary file 1 — Figure S1. [file OCR-28-104-s001.tiff]
