# Supplementary material for: Three‐dimensional assessment on digital cast of spontaneous upper first molar distorotation after Ni‐ti leaf springs expander and rapid maxillary expander: A two‐centre randomized controlled trial
Source: Orthod Craniofac Res. 2024 Sep 8;28(1):104–15. doi: 10.1111/ocr.12849 (PMC11701968; doi:10.1111/ocr.12849)
Supplement: Supplementary file 2 — Table S1. [file OCR-28-104-s002.docx]

Supplementary Table 1. Independent sample t-test for the comparison between Leaf Expander and RME concerning the amount upper first molar of distorotation.

|  | Leaf Expander | RME | Significance |
| --- | --- | --- | --- |
|  | T1-T0  Mean ± SD | T1-T0  Mean ± SD | P value |
| D6 | 6.32±3.82 | 3.94±2.51 | **0,000** |
| D16+D26 | 12.65±4.82 | 7.88±3.91 | **0,000** |
| Rot cross-bite side | 6.42±3.65 | 3.61±2.34 | **0,000** |
| Rot no cross-bite sie | 6.25±3.95 | 4.20±2.81 | **0,000** |

D6 represents the mean of the angular values obtained from all D16 and D26 measurements;

D16+D26 indicates the sum of the distorotation of 16 and 26.

**Bold**: significant difference between groups (p value< 0.05)
